# Physiological and transcriptomic evaluation of salt tolerance in Egyptian tomato landraces at the seedling stage

**DOI:** 10.1186/s12870-025-06358-4

**Published:** 2025-04-22

**Authors:** Mohammed Ali, Fatma M. O. Aboelhasan, Ahmed Ali Abdelhameed, Fathia A. Soudy, Doaa Bahaa Eldin Darwish, ElSaka Zeinab I.M., Rasha M.A. khalil, Karima Mohamed El-Absy, Aesha H. Abdel Kawy

**Affiliations:** 1 Maryout Research Station, Genetic Resources Department, Desert Research Center, 1 Mathaf El-Matarya St, El-Matareya, Cairo, 11753 Egypt; 2 Agricultural Botany Department (Genetics), Faculty of Agriculture, Assuit Branch, Al-Azhar University, 71524 Assuit, Egypt; 3 Genetics and Genetic Engineering Department, Faculty of Agriculture, Benha University, Moshtohor, 13736 Egypt; 4 Department of Biology, Faculty of Sciences, University of Tabuk, Tabuk, 71491 Saudi Arabia; 5 Genetic and Cytology Unit, Genetic Resources Department, Desert Research Center, 1 Mathaf El-Matarya St, El-Matareya, Cairo, 11753 Egypt; 6 6 Ecophysiology Unit, Plant Ecology and Range Management Department, Desert Research Center, 1 Mathaf El-Matarya St., El-Matareya, Cairo, 11753, Egypt, Cairo, Egypt

**Keywords:** *Solanum lycopersicum*, The expression cube tool, Salinity stress, Putative tissue expression pattern, Physiological and metabolic changes

## Abstract

**Background:**

Tomato (*Solanum lycopersicum*) is an essential vegetable crop with a wonder fruit used as a good source for human food and health-promoting worldwide. Drought, water salinity, and soil salinity are the commonly known environmental factors that can limit the productivity of various crops between 30% and 50% of final yields. To counter these previous effects, scientists have focused their research on studying how tomato plants at different development stages behave under various saline environmental conditions.

**Results:**

In this study, we used bioinformatics analysis tools to identify the putative genes that are related to salt tolerance in tomatoes based on the percentage of similarity with salt tolerance genes from soybean, rice, wheat, barley, Arabidopsis and other plants. Within these, 254 genes were identified as putatively involved in salt tolerance in tomatoes. Furthermore, the putative tissue expression pattern of these genes under different times from various abiotic stresses was analyzed. Also, the Expression Cube tool was used to predict the putative expression of our target genes at various tissues in fruit development. Then we study the effect of various concentrations from Sodium chloride (NaCl) at different times on the behavior of two Egyptian tomato genotypes through estimate the physiological and metabolic changes such as; soluble sugars, glucose, fructose, total chlorophyll, chlorophyll a, and chlorophyll b contents. Moreover, the relative expression levels of salt tolerance genes in tomato *SlAAO3*,* SlABCG22*,* SlABF3*,* SlALDH22A1*,* SlAPX2*,* SlAVP1*,* SlCYP175A*,* SlNHO1*,* SlP5CS*,* SlPIP1*,* SlTPS1* and *SlUGE-1*, were investigated in both tomato genotypes under various concentrations from salt tolerance in comparison with the wild-type plants.

**Conclusions:**

At the end, bioinformatics tools help in the determination of novel genes in tomato that related with tomato plant response to salt stresses. Finally, the findings reported in this article are helpful to assess the two Egyptian tomato genotypes and for understanding the roles of candidate genes for tolerance to saline conditions. And offering insights into future using these genes for generating stress-resistant tomatoes and improving agricultural sustainability.

**Supplementary Information:**

The online version contains supplementary material available at 10.1186/s12870-025-06358-4.

## Highlights

Evaluation of salt tolerance mechanisms in two Egyptian tomato genotypes.

Putative tissue expression pattern of 254 genes under different time from various abiotic stresses.

Putative expression of our target genes at various tissues in fruit development using the Expression Cube tool.

**Supplementary Information:**

The online version contains supplementary material available at 10.1186/s12870-025-06358-4.

## Background

Salinity, cold drought, high temperature, and other stress affect development and growth in various plants, especially tomatoes. They are a major environmental factor that increases the percentage of damage in the yield and production, especially the effect of salinity, since total biomass, seed, and fruit yield are often reduced when plants were growing under salt and salinity conditions [1]. This kind of stress triggers consequences on tomato and other plants geographical growth and distribution, which is considered one of the important determinants in agricultural production [2]. Moreover, plants can be divided into two classes based on the ability of the plant to grow and acclimatize to salinity; salt tolerance (ST), and salt sensitivity (SS). Salt tolerance represents the likability of a plant to respond and grow under salt stress, and salt tolerance is an induced response where plants acquire an increase of salt stress acclimation upon a prior low but non-toxic salt stress treatment [3]. And the secret behind the ability of some plants to be ST is the content of this plant, which is related to various mutagenic and quantitative traits that are related to metabolic changes in comparison with the SS plants [4].In addition, several reports have demonstrated the roles of metabolites in various plant response to a specific stress [5–9]. For example, various sugars, carotenoids, total chlorophyll, chlorophyll a, chlorophyll b and lipids are induced by different types of abiotic stresses in different plant species [5, 10].In this context, acquired ST involves major physiological and metabolic changes, which are related to the extensive reprogramming of gene expression [11]. On the other hand, these previous changes affect various plant processes, such as cell wall and vacuole composition, growth, photosynthesis, osmotic regulation of cell membranes, plant hormone signaling, various carbohydrates catabolism and anabolism [12].

Tomatoes (*S. lycopersicum* L.) belong to the Solanaceae family. It is a scrambling subshrub and valuable vegetable horticulture plant that is native to Peru and is widely cultivated in more than one hundred and fifty countries with moderate and tropical climates across the globe, particularly in Egypt, Jordan, East European Russia, Mexico Northwest, Taiwan, India, West Asia, Central African Republic, United States of America, East Asia, and other worldwide countries as listed in the Royal Botanic Gardens database (https://powo.science.kew.org/taxon/urn:lsid:ipni.org:names:316947-2). *S. lycopersicum* is widely consumed worldwide as fresh fruit or processed food products for their active components, e.g., carotenoids, vitamin C, vitamin E, quercetin, kaempferol, proteins, naringenin, β-sitosterol, caffeic, ferulic, and essential amino acids [13–16].

This study aimed to (i) Genetic and physiological behavioral assessment for two Egyptian tomato genotypes (Super Strain B and Edkawy); (ii) examine the effect of different concentration of NaCl salt stress on seed at germination and seedling stage of previous two genotypes; (iii) Selection and predict the candidate genes that related with salt tolerance; (iv) Predict the putative tissue expression pattern of our target genes under different time and various tissues in *S. lycopersicum* M82 Fruit Development; (v) Evaluation of the effect of different concentrations of salinity on different metabolic levels; (vi) QRT-PCR of highly expressed genes that related to salt tolerance. The combination of bioinformatics analysis with physiological, metabolic analysis, qPCR analyses, and putative expression patterns allows us to identify candidate genes that are linked with salt tolerance in Egyptian tomato genotypes. Finally, we can consider these previously identified genes key genes related to salt tolerance responses in tomato.

## Results

### Morphological changes and germination percentage of two tomato genotypes under salinity stress

**Fig. 1:**
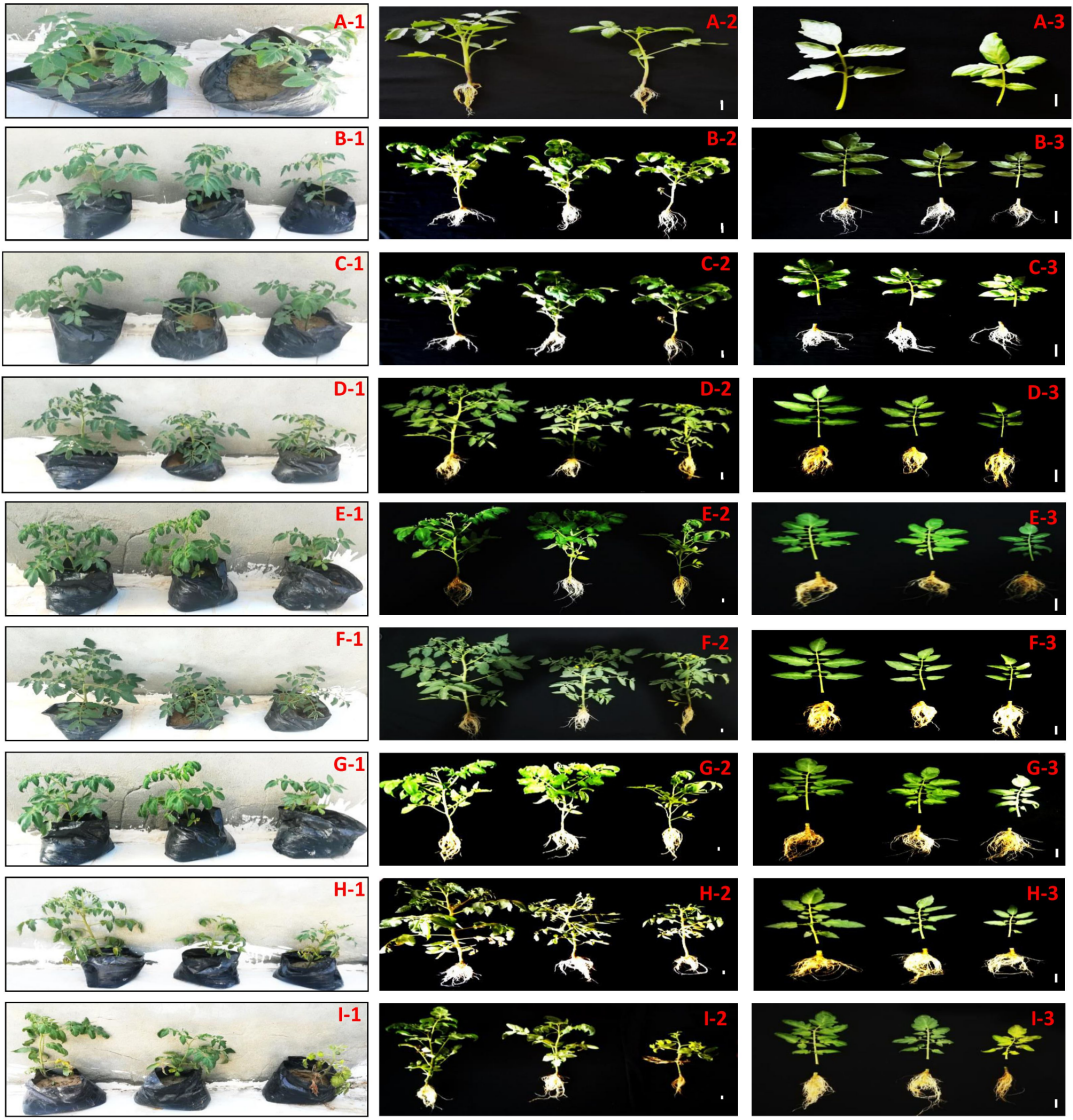
Effect of various concentration from NaCl on the growth and development of two Egyption tomato genotypes (Edkawy and Super strain B). (A-1 and I-1) Egyption tomato genotypes before and under different concentration from NaCl stress. (A-2 and I-2) Root and shoot phenotypes of tomato genotypes before and under different concentration from NaCl stress. (A-3 and I-3) Root and shoot phenotypes of tomato genotypes before and under different concentration from NaCl stress. Scale bar = 5 cm

Salinity strongly influences all the aspects of a tomato plant’s life, producing changes even in the morphological characteristics. So in this experiment we assess the behaviour of two Egyptian tomato genotypes under various concentrations of salinity using NaCl. And from our results, we found the morphological appearance of these two has been affected by different concentrations of salinity at different times at different development stages; see Fig. 1. On the other hand, we study the effect of various concentrations of NaCl on the germination rate of these two tomato genotypes seeds. And both genotypes showed a delay in seed germination, with germination percentage about 66.66 and 33.33% under 2500 and 5000 ppm from NaCl in Edkawy and with germination percentages of about 33.33% and 33.33% under 2500 and 5000 ppm from NaCl in Super strain B, in comparison with the control with a germination percentage of 100%.

### Identification, annotation, and functional classification of genes associated with salt tolerance in tomato genomics

Bioinformatics tools and databases have emerged as scientific tools for the identification, annotation, and functional classification of various genes related to different biotic and abiotic stresses and metabolism in many model and non-model plants. For that, we used genes related to salt tolerance from soybean, rice, wheat, barley, Arabidopsis and other plants as a query to hunt the genes that have the same function in the *S. lycopersicum* genomics; see Supplementary Table 1. Based on sequence homology, we identified 254 genes that are putatively involved in salt tolerance and other abiotic stress in tomatoes. Moreover, some of these genes are transcription factors (TF) such as; SlABF3 (Solyc01g108080, Solyc10g050210 and Solyc11g044560), SlAP2 (Solyc06g050520 and Solyc05g052410), SlCBF4 (Solyc03g026270, Solyc03g026280, Solyc03g124110, Solyc03g026270, Solyc03g026280 and Solyc03g124110), SlCpMYB10 (Solyc01g057910 and Solyc05g053330), SlHARDY (Solyc10g083560 and Solyc09g009240), SlJERF1 (Solyc03g123500, Solyc12g049560 and Solyc06g063070), SlMYB44 (Solyc02g092930 and Solyc02g092930), SlMYB60 (Solyc10g081490), SlMYC2 (Solyc08g076930), SlbZIP23 (Solyc04g078840) and SlWRKY45 (Solyc09g015770 and Solyc03g095770), Which encoded to ABA responsive element (ABRE) binding bZIP factor, AP2 domain TF, DREB family TF, MYB TF, AP2/ERF-like transcription factor, Ethylene response factor (ERF), MYB 44 type TF, R2R3-MYB, transcriptional activator of ABA signaling, bZIP23 transcription factor and WRKY type TF, respectively see Supplementary Table 1.

### Putative tissue expression pattern of *S. lycopersicum* salt tolerance genes under different times from a biotic stress based on *Arabidopsis thaliana* transcript expression

**Fig. 2:**
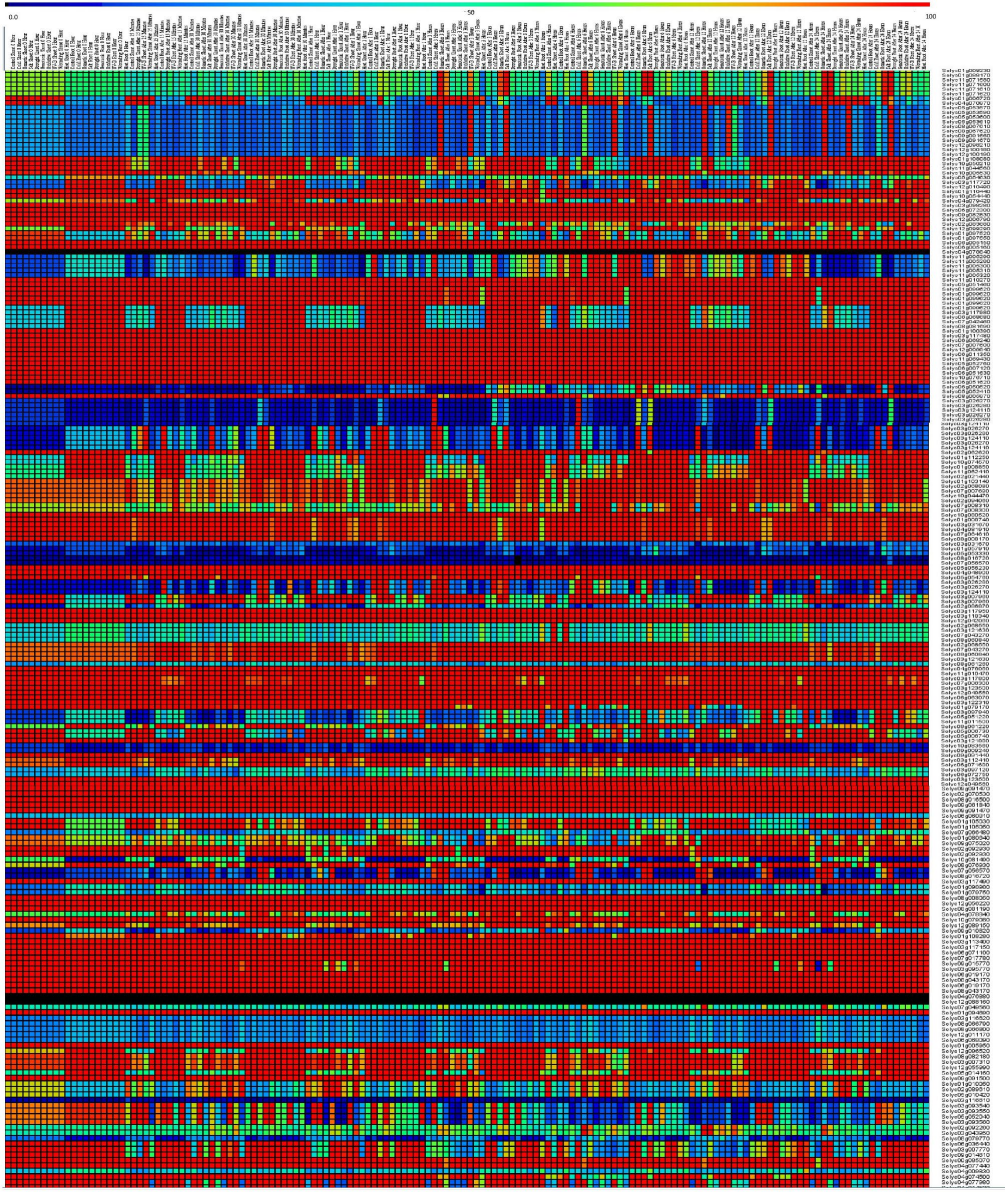
Heat map represent the putative tissue expression pattern of *S. lycopersicum* salt tolerance genes under different times from a biotic stress based on *Arabidopsis thaliana* transcript expression, The color represents the expression scale (the more intense the red color, the more gene expression)

The putative tissue expression of our candidate salt tolerance genes from tomato based on *A. thaliana* was analysed to understand their roles and functions in salt tolerance and other a biotic stress (Fig. 2). The results reported that some of our salt tolerance genes from *S. lycopersicum* were highly expressed in various tissues under different a biotic stress. Forexample, the *SlABC* (Solyc01g110440 and Solyc10g054440), *SlAPX2* (Solyc06g005150 and Solyc06g005160), *SlHB6* (Solyc11g010270 and Solyc05g051460), *SlAVP1* (Solyc01g100390, Solyc03g117480, Solyc06g068240, Solyc07g007600 and Solyc12g009840), *SlPIP1* (Solyc06g011350 and Solyc11g069430), *SlPtdIns-PLC2* (Solyc05g052760, Solyc06g007120, Solyc06g051630, Solyc10g076710 and Solyc06g051620), *SlCOIN* (Solyc10g080520), *SlCRT* (Solyc05g056230 and Solyc04g048900), *SlERD1* (Solyc03g117950, Solyc03g118340 and Solyc12g042060), *SlGF14lambda* (Solyc04g076060 and Solyc11g010470), *SlERF3* (Solyc03g123500, Solyc12g049560 and Solyc06g063070), *SlTP55* (Solyc03g122310), *SlHDA19* (Solyc09g091440), *SlJERF1* (Solyc03g123500, Solyc12g049560 and Solyc06g063070), *SlKAT2* (Solyc02g070530, Solyc08g016500 and Solyc09g061840), *SlAQP1* (Solyc08g008050, Solyc12g056220 and Solyc08g081190), *SlT2* (Solyc03g113400, Solyc03g117150, Solyc06g071100 and Solyc07g017780), *SlP5CS* (Solyc06g019170 and Solyc08g043170), *SlP5CS1* (Solyc06g019170 and Solyc08g043170), *SlPIP1* (Solyc01g094690), *SlPP2Ac-1* (Solyc01g005950) and *SlSAD1* (Solyc09g091500) genes were highly expressed in most of tissues under different a biotech stress (e.g. cold, drought, genotoxic, salt, osmotic, oxidative, UV-B, Wounding, Heat), especially salt stres at various times, such as; Control Shoot 0 h, Salt Shoot 0 h, Salt Root 0 h, Salt Shoot After 30 min, Salt Root After 30 min, Salt Shoot After 1 h, Salt Root After 1 h, Salt Shoot After 3 h, Salt Root After 3 h, Salt Shoot After 6 h, Salt Root After 6 h, Salt Shoot After 12 h, Salt Root After 12 h, Salt Shoot After 24 h, Salt Root After 24 h Fig. 2 and Supplementary Table 2). Moreover, these previous genes encoding to arginine decarboxylase and polyamine biosynthesis; ascorbate peroxidase 2, H2O2 scavenger; homeodomain protein, target of ABI1; vacuolar membrane H+-Pyrophosphatase; plasma membrane intrinsic proteins; phosphatidylinositol-specific phospholipase C2; RING-finger protein; Calreticulin, Ca2 + binding protein; chloroplast-targeted Clp protease reg SU; 14-3-3 protein; AP2/ERF-like transcription factor; stress-induced plant antiquitin-like protein; histone deacetylase; Ethylene response factor (ERF); 3-ketoacyl-CoA thiloase-2; PIP1 plasma membrane aquaporin; plasma membrane proton ATPase; Pyrroline-5-Carboxylate Synthase (glutamtate proline); plasma membrane intrinsic proteins (PIPs); PP2A, catalytical subunit and Supersensitive to ABA and drought 1, respectivelly (Fig. 2 and Supplementary Table 2).

### The putative expression of our target genes at various tissues in fruit development using the expression cube tool

**Fig. 3:**
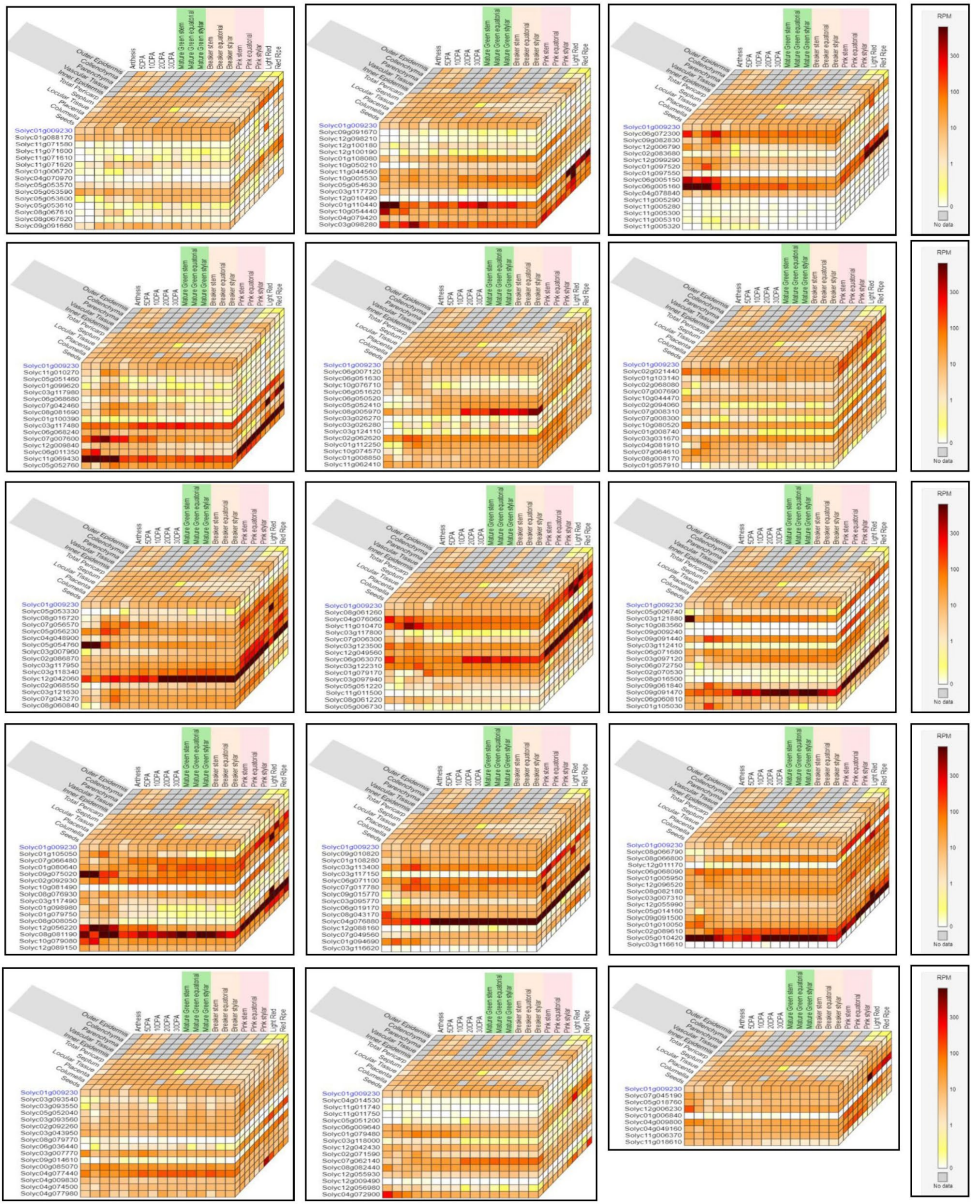
The putative expression of our target genes in various tissues in fruit development using the Expression Cube tool. The color represents the expression scale (the more intense the red color, the more gene expression)

The Expression Cube tool was used to predict the putative expression of our target genes in various tissues in fruit development. The results showed that most of our salt tolerance genes from *S. lycopersicum* were highly expressed in all eleven tissues (e.g. Outer Epidermis, Collenchyma, Parenchyma, Vascular Tissue, Inner Edidermis, Total pericarp, Septum, Locular Tissue, Placenta, Columella and Seeds) in combine with another sixteen tissues (e.g. Anthesis, 5DPA, 10DPA, 20DPA,30DPA, Mature Green Stem, Mature Green equatorial, Matuer Green Stylar, Breaker Stem. Breaker equatorial, Breaker Stylar, Pink Stem, Pink equatorial, Pink Stylar, Light Red and Red Ripe) at fruit development. For example, *SlAAO3* (Solyc01g009230), *SlABCG40* (Solyc05g053590), *SlABC* (Solyc10g054440), *SlAGO1*(Solyc03g098280), *SlAPX2* (Solyc06g005150 and Solyc06g005160), *SlAVP1* (Solyc03g117480 and Solyc07g007600), *SlPIP1* (Solyc11g069430), *SlCAU1*(Solyc08g005970), *SlCBP20* (Solyc02g062620), *SlCIPK03* (Solyc02g021440), *SlCOIN* (Solyc10g080520), *SlCPK21* (Solyc03g031670), *SlCRT* (Solyc05g056230), *SlDHAR2* (Solyc05g054760), *SlERD1* (Solyc03g117950, Solyc03g118340 and Solyc12g042060), *SlGF14lambda* (Solyc04g076060 and Solyc11g010470), *SlERF3* (Solyc03g123500, Solyc12g049560 and Solyc06g063070), *SlTP55* (Solyc03g122310), *SlHAB1* (Solyc03g121880), *SlHDA19* (Solyc09g091440), *SlHDA6* (Solyc06g071680 and Solyc03g097120), *SlKAT2* (Solyc09g061840), *SlMRP4* (Solyc01g080640 and Solyc09g075020), *SlMYB44* (Solyc02g092930), *SlMYC2* (Solyc08g076930), *SlAQP1* (Solyc12g056220 and Solyc08g081190), *SliSAP8* (Solyc10g079080), *SlST2* (Solyc03g113400 and Solyc06g071100), *SlPEPCK* (Solyc04g076880), *SlPLD* (Solyc08g066800 and Solyc06g068090), *SlSAMDC* (Solyc02g089610 and Solyc05g010420), *SLAH3* (Solyc03g007770), *SlSQE1* (Solyc00g085070 and Solyc04g077440), *SlbZIP1*(Solyc01g079480), *SlWXP1* (Solyc04g072900), *SlXERICO* (Solyc12g006230) and *SlCPK4* (Solyc04g009800, Solyc04g049160 and Solyc10g074570) genes were highly expressed in all tissues at fruit development (Fig. 3). Furthermore, these previous genes encoding Arabidopsis aldehyde oxidase, catalyzes final step in ABA biosynthesis; Arginine decarboxylase; Argonaute1; Ascorbate peroxidase 2, H2O2 scavenger; vacuolar membrane H+-Pyrophosphatase; Plasma membrane intrinsic proteins; H4R3sme2 (for histone H4 Arg 3 with symmetric dimethylation)-type histone methylase protein arginine methytransferase5/Shk1 binding protein1; Cap Binding Protein 20 (mRNA cap); calcineurin B-like protein-interacting protein kinase; RING-finger protein; calcium-dependent protein kinase; Calreticulin, Ca2 + binding protein; dehydroascorbate reductase; chloroplast-targeted Clp protease reg SU; 14-3-3 protein; AP2/ERF-like transcription factor; stress-induced plant antiquitin-like protein; Protein phosphatases of the 2 C family (PP2C); histone deacetylase; Heat shock transcription factor; 3-ketoacyl-CoA thiloase-2; multidrug resistance-associated protein, ABC transporter; MYB type TF; transcriptional activator of ABA signaling; PIP1 plasma membrane aquaporin; Stress associated protein gene family, zinc finger domain; plasma membrane proton ATPase; PEP carboxykinase; Phospholipase D, lipid degrading enzyme; S-adenosyl methionine decarboxylase, Polyamin synthesis; Guard cell S-type anion channel (SLAC1 homolog); Squalene epoxidase1; bZIP TF; putative AP2 domain-containing TF; activates wax production in the acyl-reduction pathway; Small protein, N-term- TM domain and RING-H2 zinc-finger motif and Calcium-dependent protein kinase, respectively see (Fig. 3).

### Differential gene expression by qRT-PCR

**Fig. 4:**
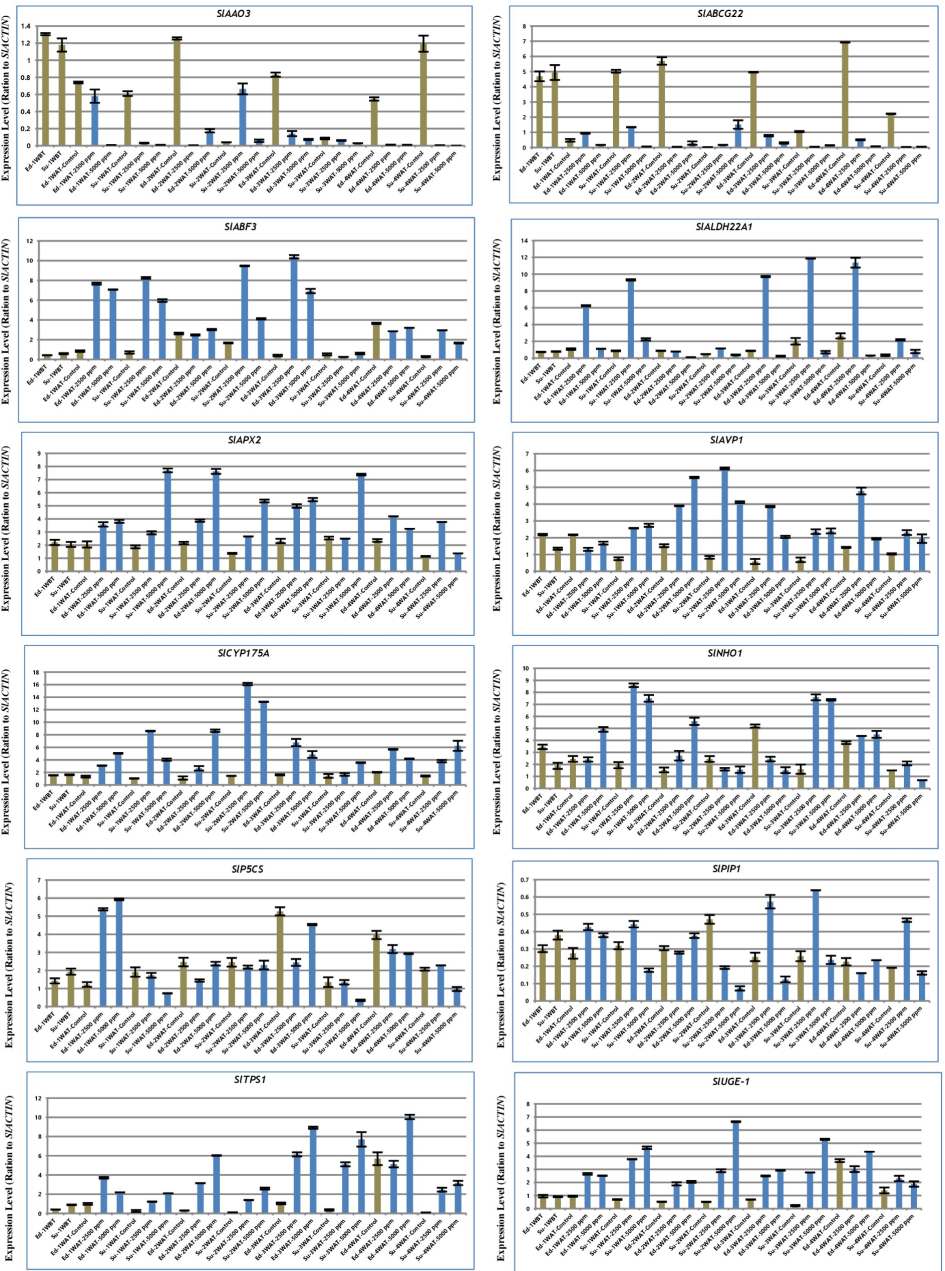
Quantitative RT-PCR of genes related with salt tolerance in tomato. The relative expressions of *SlAAO3*,* SlABCG22*,* SlABF3*,* SlALDH22A1*,* SlAPX2*,* SlAVP1*,* SlCYP175A*,* SlNHO1*,* SlP5CS*,* SlPIP1*,* SlTPS1* and *SlUGE-1* were calculated. The values are means ± SE of three biological replicates. Ed (Edkawy), Su (Super strain B), WBT (Week Before treatment), WAT (Week After Treatment)

To elucidate the expression levels for twelve candidate genes related to salt tolerance in tomato (Supplementary table Tables S3 and Fig. 4) across different concentrations from salt treatments and control conditions. The relative expression levels of our twelve genes, including *SlAAO3*,* SlABCG22*,* SlABF3*,* SlALDH22A1*,* SlAPX2*,* SlAVP1*,* SlCYP175A*,* SlNHO1*,* SlP5CS*,* SlPIP1*,* SlTPS1* and *SlUGE-1*, were detected. For instance, *SlAAO3* and *SlABCG22* showed the lowest expression levels in both tomato genotypes under various concentrations of salt tolerance compared with controls. Moreover, the expression level of *SlABF3* was highest in Ed-3WAT-2500 ppm, followed by Su-2WAT-2500 ppm, Su-1WAT-2500 ppm, Ed-1WAT-2500 ppm, and Ed-1WAT-5000 ppm in comparison with the control. Furthermore, the expression levels of *SlALDH22A1* was highest in Su-3WAT-2500 ppm, followed by Ed-4WAT-2500 ppm, Ed-3WAT-2500 ppm, Su-1WAT-2500 ppm and Ed-1WAT-2500 ppm in comparison with controls. *SlAPX2* transcription levels were markedly increased in Su-1WAT-5000 ppm then Ed-2WAT-5000 ppm, Su-3WAT-5000 ppm and Ed-3WAT-5000 ppm. While the highest expression levels of *SlAVP1* gene was reported in Su-2WAT-2500 ppm, Ed-2WAT-5000 ppm, Ed-4WAT-2500 ppm, Su-2WAT-5000 ppm, Ed-2WAT-2500 ppm and Ed-3WAT-2500 ppm. Also, *SlCYP175A* gene was highly expressed in Su-2WAT-2500 ppm, Su-2WAT-5000 ppm, Su-1WAT-2500 ppm, Ed-2WAT-5000 ppm and Ed-3WAT-2500 ppm. In addition, the highest expression levels of *SlNHO1* gene was detected in Su-1WAT-2500 ppm, followed by Su-3WAT-2500 ppm, E Su-1WAT-5000 ppmd-2WAT-5000 ppm, Ed-2WAT-5000 ppm. Besides, *SlP5CS* expression was highest in Ed-1WAT-2500 ppm and Ed-1WAT-5000 ppm. On the other hand, *SlPIP1* transcription was highest in Su-3WAT-2500 ppm and Ed-3WAT-2500 ppm. The highest expression level for SlTPS1 was observed in Ed-4WAT-5000 ppm, Ed-3WAT-5000 ppm, Su-3WAT-5000 ppm, Ed-3WAT-2500 ppm and Ed-2WAT-5000 ppm. Finally, the expression level of *SlUGE-1* was highest in Su-2WAT-5000 ppm, Su-3WAT-5000 ppm, Su-1WAT-5000 ppm, Ed-4WAT-5000 ppm, Su-1WAT-2500 ppm, and Su-1WAT-2500 ppm in comparison with the control; see Fig. 4. In the context, we found the previous salt tolerance genes have variable expression levels in both tomato genotypes at different stress times and under salinity and non-salinity conditions. These results suggest that these genes may play key roles in responses to salt stresses in tomato.

### Salt stress alters various physiological and biochemical contents in tomato genotypes

**Fig. 5:**
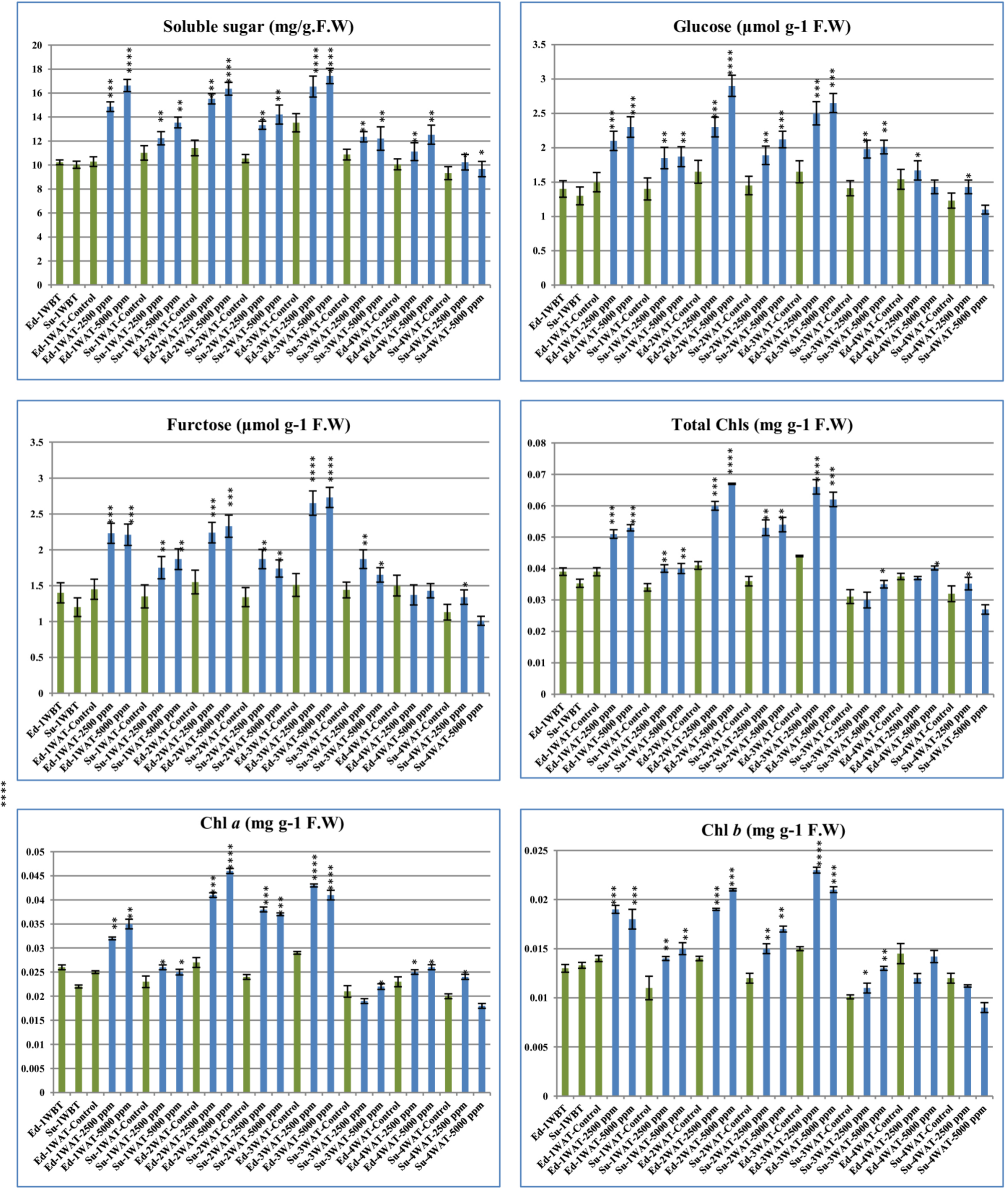
Analysis of physiological and biochemical parameters from two Egyptian tomato genotypes before and after treatment with various concentrations from NaCl. Ed (Edkawy), Su (Super strain B), WBT (Week Before Treatment), WAT (Week After Treatment), Significance levels were indicated as(*) for P-values less than 0.05, (**) forP < 0.01, and (***) for *P* < 0.001

Sugars and soluble sugars serve as the primary carbon source for various key pathways involved in different primary and secondary metabolites. The levels of soluble sugars, glucose, fructose, total chlorophyll, chlorophyll a, and chlorophyll b contents were assessed in Super Strain B and Edkawy genotypes under various concentrations from salinity stress. Results indicated higher levels of these components in most of the treatments compared to the control (Fig. 5). Forexample, the level of soluble sugar content increased in all genotypes compared with the control. Similarly, average glucose contents visually increased in all genotypes at various concentrations in compared with control except the Su-4WAT-5000 ppm (see Fig. 5). Moreover, the fructose levels showed respective increases in all genotypes treated under most concentration compared with the control, beyond the Ed-4WAT-2500 ppm and Su-4WAT-5000 ppm. Furthermore, the total chlorophyll (Total Chls) contents were reported to be the highest at all genotype treatments unless Su-3WAT-2500 ppm, Ed-4WAT-2500 and Su-4WAT-5000 ppm were compared with the control. Additionally, average chlorophyll a (Chl a) level increased in all genotype treatments compared with the control, except the Su-3WAT-2500 ppm and Su-4WAT-5000 ppm. At the end, average chlorophyll b (Chl b) level increased in all genotype treatments compared with the control, except the Ed-4WAT-2500 ppm, Su-4WAT-2500 ppm and Su-4WAT-5000 ppm.

## Discussion

Most plants in the Solanaceae family in general and the Solanum genus in particular have a strong savoury umami flavor due to their content of various vitamins, dietary fibre, fat, carbohydrates, sugars, minerals, and pigments; for that, the fruit is used as a good source for human food and health-promoting worldwide [17]. Tomato growth and production are affected by natural climatic changes, especially biotic stresses such as cold, drought, high temperatures, water fluoridation, and salinity [18]. So in this study we evaluated the vegetative growth and optical morphological changes of two Egyptian tomato genotypes (Edkawy and Super strain B) under various concentrations of NaCl stress. And from our results, we found that both Egyptian tomato genotypes have been noticeably affected by the various concentrations of NaCl at different development stages, especially the genotype Super strain B, which was affected by all concentrations at all development stages, and we can note and perceive these through the different changes at the vegetative growth and morphological levels (see Fig. 1), and the same results about the effect of salinity and salt stress on tomato plants have been reported by several investigators [19–21]. Moreover, we investigate the effect of sodium chloride (NaCl) on both genotype seed germination, and we found the various NaCl concentrations delay the seed germination, which means the NaCl has a affect on the seed germination process, and these results are in line with [12]. In this context and based on the above, the reason behind the effect of different concentrations of salinity on the growth and alteration of phenological development of tomato plants can be attributed to the effects of salinity on the vital processes in the plant, which are represented in the excessive uptake of Na + and chloride ions (cytotoxicity) with time, which lead to physiological dysfunctions, affect photosynthesis, destabilise membranes, respiration, mineral nutrient homeostasis, starch metabolism, absorb the water, and nitrogen fixation [2, 22, 23].

In this context, studies conducted in the last decade have highlighted that the response of tomato plants to salinity depends mainly on the genotype and several gene families [14–16, 24–27]. Furthermore, this study employed bioinformatic tools to identify and predict key genes that represented various pathways in *S. lycopersicum* genomics, revealing 254 genes encompassing various metabolic pathways (see Supplementary Tables 1 and Fig. 2). Gene ontology analysis showed that these previous genes are potentially involved in many biological functions that are related to the ability of tomatoes to tolerate salt and other abiotic stress. Forexample, some of these genes are transcription factors (TF) such as; *SlABF3* (Solyc01g108080, Solyc10g050210 and Solyc11g044560), *SlAP2* (Solyc06g050520 and Solyc05g052410), *SlCBF4* (Solyc03g026270, Solyc03g026280, Solyc03g124110, Solyc03g026270, Solyc03g026280 and Solyc03g124110), *SlCpMYB10* (Solyc01g057910 and Solyc05g053330), *SlHARDY* (Solyc10g083560 and Solyc09g009240), *SlJERF1* (Solyc03g123500, Solyc12g049560 and Solyc06g063070), *SlMYB44* (Solyc02g092930 and Solyc02g092930), *SlMYB60* (Solyc10g081490), *SlMYC2* (Solyc08g076930), *SlbZIP23* (Solyc04g078840) and *SlWRKY45* (Solyc09g015770 and Solyc03g095770), Which encoded to ABA responsive element (ABRE) binding bZIP factor [28], AP2 domain TF [29], DREB family TF [30], MYB10 TF [31], AP2/ERF-like transcription factor ( [32], Ethylene response factor (ERF) [33], MYB 44 type TF [34], R2R3-MYB [35], transcriptional activator of ABA signaling [36], bZIP23 transcription factor [37] and WRKY type TF [38], respectively see Supplementary Table 1.

In addition, the analysis performed using the Arabidopsis eFP browser tool allowed us to predict the putative tissue expression pattern of *S. lycopersicum* salt tolerance genes under different times from abiotic stress based on *A. thaliana* transcript expression (Fig. 2). This analysis showed that these 254 genes had variable expression in all tissues under different abiotic stress. While some genes from these 254 genes were highly expressed in some tissues under different abiotic stress (Fig. 2 and Supplementary Table 1). Various studies have shown that the genes mentioned below were highly expressed in plants when exposed to different concentrations of NaCl, such as; *SlABC* and *SlCOIN* from rice [39, 40], *SlAVP1*,* SlAPX2*,* SlHB6*,* SlPIP1*,* SlERD1*,* SlHDA19*,* SlKAT2*,* SlT2*,* SlP5CS* and *SlSAD1* from *A. thaliana* [41–49], *SlPtdIns-PLC2* from *Brassica napus* [50], *SlCRT* and *SlPP2Ac-1* from *Triticum aestivum* L [51, 52]., *SlGF14lambda* from cotton (Yan et al., 2004), *SlERF3* and *SlTP55* from soybean [53, 54], *SlAQP1* and *SlPIP1* from tobacco [55, 56].

On the other hand, various studies referred to the salinity effects not ceasing only at the plantlet or different development stages but the salinity affects tomato fruit development as a final product. So, in this study we used the Expression Cube tool to predict the putative expression of our target genes at various tissues in fruit development (see Fig. 3). And from our results, we found all our genes have various expression levels at all eleven tissues in combination with another sixteen tissues at fruit development. Additionally, some genes were highly expressed in all previous tissues, which means these genes can be used as markers for salt tolerance mechanisms [15, 16], (see Fig. 3 and Supplementary Table 1). In addition, it was observed that the *SlAAO3*,* SlABCG40*,* SlAGO1*,* SlAPX2*,* SlAVP1*,* SlPIP1*,* SlCAU1*,* SlCBP20*,* SlCPK21*,* SlERD1*,* SlGF14lambda*,* SlTP55*,* SlHAB1*,* SlHDA19*,* SlHDA6*,* SlKAT2*,* SlMRP4*,* SlMYB44*,* SlMYC2*,* SlST2*,* SlPEPCK*,* SLAH3*,* SlSQE1*, *SlXERICO* from Arabidopsis [34, 36, 41, 42, 44, 51, 52, 57–69], SlABC, SlCIPK03, SlCOIN, SliSAP8 from rice [39, 40, 45, 46, 47,], SlCRT from wheat [50], SlDHAR2 and SlAQP1 from tobacco [55,], SlERF3 from soybean [53], SlPLD from *Vigna unguiculata* L [70]., SlSAMDC from *Datura stramonium* [71], SlbZIP1 from *Tamarix hispida* [72], SlWXP1 from *Medicago truncatula* [73], SlCPK4 from maize [74], genes were highly expressed in all tissues at fruit development (Fig. 3).

In general, there is a relationship between salinity concentrations and the extent of response and expression of salinity tolerance genes. For example, the following genes *SlAAO3* [57], *SlABCG22* [75], *SlABF3* [28], *SlALDH22A1* [76] *SlAPX2* [42], *SlAVP1* [41], *SlCYP175A* [77], *SlNHO1* [78], *SlP5CS* [79], *SlPIP1* [44], *SlTPS1* [80] and *SlUGE-1* [40] are responding as expected, and their expression levels wavering at various development stages along the treatment (Fig. 4). In the context, the expression of *SlAAO3* and *SlABCG22* genes were low at various development stages along the treatment in compared with control, particular the *SlAAO3*, which related with aldehyde oxidase and have important role in catalyzes the final step in ABA biosynthesis. And these results are in line with several studies that reported the expression of *AAO3* gene was decreased under different stress (e.g., salt and drought ) in comparison with the control plant [81–85]. While the remaining genes recorded high expression in most tissues and under different salinity concentrations in comparison with control (Fig. 4). Interestingly, the transcript levels of these previous genes are in very similar line with the putative expression data under different time from a biotic stress based on *A. thaliana* transcript expression and the putative expression at various tissues in fruit development, which was obtained from our analysis using bioinformatic tools. These previous results indicate that these genes (e.g., *SlAAO3*,* SlABCG22*,* SlABF3*,* SlALDH22A1*,* SlAPX2*,* SlAVP1*,* SlCYP175A*,* SlNHO1*,* SlP5CS*,* SlPIP1*,* SlTPS1* and *SlUGE-1*) are induced under salt stress in both tomato genotypes, suggesting the participation of these gene products in the response of tomato to salt stress at different development stages.

In comparison to tomato control plants (non-treated), salt stress treatment for tomato genotypes caused manifest differences in physiological and biochemical traits (e.g., soluble sugars, glucose, fructose, total chlorophyll, chlorophyll a, and chlorophyll b). In the context that, we found epistatic relationship between the metaolism change leves and the expression of some key genes in both tomato genotypes under different stress times. For example, Yang et al., 2014; study the role of AAO3 gene in regulatory, module and promotes of chlorophyll degradation via ABA biosynthesis in Arabidopsis leaves [86]. Considering the results obtained, we find that the expression of this gene in the control plants was high compared to the plants under salinity stress. On the other hand, we find that the levels of total chlorophyll and type A and B were high in the plants under salinity stress and low in the control, which indicates the role of this gene in the synthesis and degradation of various chlorophyll types [86]. Also, Fichtner et al., 2020; Voogd et al., 2016, and Pego and Smeekens, 2000, have been study the function role of TPS1 Gene in embryo and plant growth and development at nomal condation or under stress throught controling in the sucrose-signaling metabolite [87–89]. furthermore, we found apostive relshionship between the expression of this gene in tomato and the level of various sucrose [87–89]. In this context, the contents of all previous biochemicals were significantly higher in Super Strain B and Edkawy genotypes under various concentrations of salinity stress compared with non-stressed genotypes (control) (Fig. 5), which means the level of biochemical contents was affected by salt stress and affects the various metabolic pathways in which these components share and this explains the changes in morphological and physiological characteristics of plants under salt stress. Furthermore, these previous results are in line with [90–92], they found the salt stress increased the various biochemical contents in various plants such as tomato, rice, and sweet sorghum.

## Conclusions

Under normal environmental conditions, plants grow, develop, and produce without any limitation. While in a non-natural environment, which results from climatic changes and various stresses, the plant growth and development will be significantly impaired. So, there was a need to search for new genotypes with high production and ability for various stress tolerances. For that reason, the tolerance pattern of some tomato genotypes to various stressors particularly salt stress, is attributed to the dominant one and the activation of various genes that are related to salt tolerance. In this context, there is a need to understand the key mechanism through study of the function and pathways of the genes that are related to salt tolerance using various bioinformatics tools and databases. In our study, we employed bioinformatic tools and databases to identify the putative genes that are related with salt tolerance in tomato genomics. Utilising Bioinformatic databases and analysis tools, we obtained 254 genes involved in salt tolerance in tomatoes. Our putative tissue expression and the expression analysis results refer to most of these previous genes being highly expressed in all tissues at various development stages and under different abiotic stress. In addition, results showed that various concentrations from salt stress have negative effects on the growth and development of both Egyptian tomato genotypes with varied proportions. Also, salt stress has a significant effect on various physiological and biochemical characteristics of genotypes.Our results reveal that most of these genes have essential roles in tomato growth and development under salt stress. At the end, our study appended valuable information about the reason behind the ability of some genotypes against salt stress, and these results refer to how we can maximise the benefit of these genes for improving tomato plants and increasing production under stress.

## Materials and methods

### Plant material, growth condition, treatments and sampling

Two tomato genotypes were collected from Maryout Research Station, Desert Research Centre (DRC), Alexandria Governorate, Egypt: the salt-sensitive cultivar, “Super Strain B” and the salt-resistance cultivar, “Edkawy“, these two genotypes were provided by Dr. Zeinab I. El-Saka, Assoc. Prof. at the Plant Breeding Unit, Genetic Resources Department, DRC, and were used in our present study. Seeds from each genotype were sown in a plastic tray (30 × 60 cm) which contained artificial soil (perlite mixed with vermiculite) then the tray was irrigated with tap water and covered by a plastic cover and kept in the open greenhouse with normal environmental conditions near to College of Agriculture, Asuit University, and kept for one week until the seed germination. After one week, the plastic cover was removed to let the seedling grow for another week. Then the individual plantlets that were two weeks old were transplanted into plastic pots (20 × 25 cm) containing about 3kg from 1:1 clay and sand mixture, and the plantlets in plastic pots were irrigated twice a week with tap water supplied with NPK as a fertilized solution for three weeks. After three weeks of growth, three salinity (NaCl) treatments were initiated as follows: 2500 ppm, 5000 ppm, and control (only tap water) for one, two, three, and four weeks. And each plastic pot has one plantlet with five replicates and is irrigated twice a week by 0.6 L from salinity or tap water. For measurement of the biochemical contents and analysis of the gene expression levels, twenty-six samples were collected before and after all the treatments. Furthermore, all the samples are listed as follows: Ed-1WBT, Su-1WBT, Ed-1WAT-Control, Ed-1WAT-2500 ppm, Ed-1WAT-5000 ppm, Su-1WAT-Control, Su-1WAT-2500 ppm, Su-1WAT-5000 ppm, Ed-2WAT-Control, Ed-2WAT-2500 ppm, Ed-2WAT-5000 ppm, Su-2WAT-Control, Su-2WAT-2500 ppm, Su-2WAT-5000 ppm, Ed-3WAT-Control, Ed-3WAT-2500 ppm, Ed-3WAT-5000 ppm, Su-3WAT-Control, Su-3WAT-2500 ppm, Su-3WAT-5000 ppm, Ed-4WAT-Control, Ed-4WAT-2500 ppm, Ed-4WAT-5000 ppm, Su-4WAT-Control, Su-4WAT-2500 ppm and Su-4WAT-5000 ppm. And these previous symbols and abbreviations, ‘’Ed, Su, 1WBT, 1WAT, 2WAT, 3WAT and 4WAT’’ refer to ‘’ Edkawy, Super Strain B, one week before treatment, one week after treatment, two weeks after treatment, three weeks after treatment, and four weeks after treatment’’. And all these previous samples were collected and stored at -20°C until analysis.

### Seed germination test

Tomato seeds of two genotypes (Edkawy and Super strain B) were surface- sterilized for 20 min. in 30% (V/V) commercial Clorox solution (5.05% sodium hypochlorite). After that they were rinsed three times in sterile deionized water. The tomato seeds were germinated as three seeds in glass jars containing MS media with ten replicas, the components of the MS medium are; 4.3 g/L with vitamins Murashige and Skoog (1962) and 30 g/L sucrose, 100 mg/L myo- inositol, and 3 g/L Sigma phytagel, the pH of the medium was adjusted to 5.6 by KOH before adding the phytagel. The seeds were germinated in a glass Jars on seed germination medium without NaCl (control media) and with two concentrations from NaCl (2500 and 5000 ppm), the jars were incubated for three days at 25 °C in the dark, and then transferred to the growth room chamber under the following conditions: 25 °C, cool white light, photoperiod 16 h light and 8 h dark for 7 days for studying the effect of NaCl on the germination percentage of two accessions of tomato (Edkawy and Super strain B). The seed germination percentage was calculated after one week from seed culture.

### Identification and functional assignments for genes associated with salt tolerance in tomato

Putative sequences of salt tolerance genes from soybean, rice, wheat, barley, Arabidopsis, and other plants were used as a query to search against the *S. lycopersicum* genomics using blastx (search protein subjects using a translated nucleotide query) at NCBI genomics (https://blast.ncbi.nlm.nih.gov/Blast.cgi?PROGRAM=blastx%26;PAGE_TYPE=BlastSearch%26;LINK_LOC=blasthome, accessed on 15 April 2023). And the candidate genes were selected based on the high percentage of identity (% identity). After that, the alignment sequence was compared using Phytozome and KEGG databases to predict the biological function for these selected candidate genes that are associated with salt tolerance in tomato. At the end, we get 254 genes that are highly related to salt tolerance in tomato after removing the unknown and redundant genes [14, 15].

### Putative tissue expression pattern of our target genes under different times from a biotech stress based on *A. thaliana* transcript expression

Putative tissue-specific expression profiles of 254 genes from *S. lycopersicum* were extracted based on *A. thaliana* transcript expression database from 154 shoots and roots under various biotech stresses at different times, including; Control Shoot 0 h, Cold Shoot 0 h, Osmotic Shoot 0 h, Salt Shoot 0 h, Drought Shoot 0 h, Genotoxic Shoot 0 h, Oxidative Shoot 0, Hour UV-B, Shoot 0 h, Wounding Shoot 0 h, Heat Shoot 0 h, Control Root 0 h, Cold Root 0 h, Osmotic Root 0 h, Salt Root 0 h, Drought Root 0 h, Genotoxic Root 0 h, Oxidative Root 0 h, UV-B Root 0 h, Wounding Root 0 h, Heat Root 0 h, Control Shoot After 15 min, Drought Shoot After 15 min, UV-B Shoot After 15 min, Wounding Shoot After 15 min, Heat Shoot After 15 min, Control Root After 15 min, Drought Root After 15 min, UV-B Root After 15 min, Wounding Root After 15 min, Heat Root After 15 min, Control Shoot After 30 min, Cold Shoot After 30 min, Osmotic Shoot After 30 min, Shoot After 30 min, Drought Shoot After 30 min, After 30 min, Wounding Shoot After 30 min, Heat Shoot After 0 min, Control Root After 30 min, Cold Root After 30 min, Osmotic Root After 30 min, Salt Root After 30 min, Drought Root After 30 min, Genotoxic Root After 30 min, Oxidative Root After 30 min, UV-B Root After 30 min, Wounding Root After 30 min, Heat Root After 30 min, Control Shoot After 1 h, Cold Shoot After 1 h, Osmotic Shoot After 1 h, Salt Shoot After 1 h, Drought Shoot After 1 h, Genotoxic Shoot After 1 h, Oxidative Shoot After 1 h, UV-B Shoot After 1 h, Wounding Shoot After 1 h, HeatShoot After 1 h, Control Root After 1 h, Cold Root After 1 h, Osmotic Root After 1 h, Salt Root After 1 h, Drought Root After 1 h, Genotoxic Root After 1 h, Oxidative Root After 1 h, UV-B Root After 1 h, Wounding Root After 1 h, Heat Root After 1 h, Control Shoot After 3 h, Cold Shoot After 3 h, Osmotic Shoot After 3 h, Salt Shoot After 3 h, Drought Shoot After 3 h, Genotoxic Shoot After 3 h, Oxidative Shoot After 3 h, UV-B Shoot After 3 h, Wounding Shoot After 3 h, Heat Shoot After 3 h, Control Root After 3 h, Cold Root After 3 h, Osmotic Root After 3 h, Salt Root After 3 h, Drought Root After 3 h, Genotoxic Root After 3 h, Oxidative Root After 3 h, UV-B Root After 3 h, Wounding Root After 3 h, Heat Root After 3 h, Control Shoot After 4 h, Heat Shoot After 4 h, Control Root After 4 h, Heat Root After 4 h, Control Shoot After 6 h, Cold Shoot After 6 h, Hours, Genotoxic Shoot After 6 h, Oxidative Shoot After 6 h, UV-B Shoot After 6 h, Wounding Shoot After 6 h, Heat Shoot After 6 h, Control Root After 6 h, Drought Root After 6 h, Genotoxic Root After 6 h, Oxidative Root After 6 h, UV-B Root After 6 h, Wounding Root After 6 h, Heat Root After 6 h, Control Shoot After 12 h, Cold Shoot After 12 h, Osmotic Shoot After 12 h, Salt Shoot After 12 h, Drought Shoot After 12 h, Genotoxic Shoot After 12 h, Oxidative Shoot After 12 h, UV-B Shoot After 12 h, Wounding Shoot After 12 h, Heat Shoot After 12 h, Control Root After 12 h, Cold Root After 12 h, Osmotic Root After 12 h, Salt Root After 12 h, Drought Root After 12 h, Genotoxic Root After 12 h, Oxidative Root After 12 h, UV-B Root After 12 h, Wounding Root After 12 h, Heat Root After 12 h, Control Shoot After 24 h, Cold Shoot After 24 h, Osmotic Shoot After 24 h, Salt Shoot After 24 h, Drought Shoot After 24 h, Genotoxic Shoot After 24 h, Oxidative Shoot After 24 h, UV-B Shoot After 24 h, Wounding Shoot After 24 h, Heat Shoot After 24 h, Control Root After 24 h, Cold Root After 24 h, Osmotic Root After 24 h, Salt Root After 24 h, Drought Root After 24 h, Genotoxic Root After 24 h, Oxidative Root After 24 h, UV-B Root After 24 h, Wounding Root After 24 h and Heat Root After 24 h see supplementary Table S2. Expression profiles were created using the Arabidopsis plant Electronic Fluorescent Pictograph Browsers (Arabidopsis eFP browsers) (http://bar.utoronto.ca/eplant_Arabidopsis/) accessed on 5 April 2023 [14, 15, 93–97].

### Putative expression of our target genes at various tissues in *S. lycopersicum* M82 fruit development

The putative expressions of 254 genes from *S. lycopersicum* were examined at various fruit development tissues using the Expression Cube tool at TEA database (Tomato Expression Atlas; https://tea.solgenomics.net). Moreover, the expression module that represents the putative expression at various tissues in fruit development was built based on the *S. lycopersicum* transcript expression values in eleven tissues (e.g. Outer Epidermis, Collenchyma, Parenchyma, Vascular Tissue, Inner Edidermis, Total pericarp, Septum, Locular Tissue, Placenta, Columella and Seeds) in combine with another sixteen tissues (e.g. Anthesis, 5DPA, 10DPA, 20DPA,30DPA, Mature Green Stem, Mature Green equatorial, Matuer Green Stylar, Breaker Stem. Breaker equatorial, Breaker Stylar, Pink Stem, Pink equatorial, Pink Stylar, Light Red and Red Ripe) accessed on 5 June 2023.

### Validating the expression levels for genes related to salt tolerance in *S. lycopersicum* by qRT-PCR

To analyses the expression levels of genes related to the salt tolerance in different *S. lycopersicum* accessions under various concentrations from salt stress conditions (0.00, 2500 and 5000 ppm), twelve candidate genes were chosen. In this context, RNAs were extracted from leaves of all tomato wild-type (control) and accessions under various concentrations of NaCl (treatments) for quantitative RT-PCR. For cDNA synthesis 1 µg of RNAs was used to synthesize the first-strand cDNA using TransScript^®^ First-Strand cDNA Synthesis Super Mix kit as described by Ali et al., 2017, 2018; Hussain et al., 2017; El-ramah et al., 2022 [95, 98, 99, 100,].The expression profiles for these twelve genes were compared in leaf samples under various concentrations from salt stress to reveal their ‘transcriptional control level’, which may construe the epistatic-relationship between the level of mRNA production and the biological effect. The expression profiles of our chosen candidates: *SlAAO3*,* SlABCG22 SlABF3*,* SlALDH22A1*,* SlAPX2*,* SlAVP1*,* SlCYP175A*,* SlNHO1*,* SlP5CS*,* SlPIP1*,* SlTPS1* and *SlUGE-1* were analyzed. The SlB-ACTIN gene was used as a housekeeping gene, and all our primers were designed by IDTdna database (http://www.idtdna.com/scitools/Applications/RealTimePCR/) [92–108]. Moreover, the qRT-PCR reaction was carried out utilizing the CFX96 Dx Real-Time PCR Detection Systems, and the program implemented was executed as follows: an initial denaturation phase at 95 °C for 5 min, followed by 35 cycles of denaturation at 96 °C for 36 s, annealing at 60 °C for 30 s, and extension at 72 °C for 1.5 min (see Supplementary Table S3). For qRT-PCR, three biological replicates from leaves were sampled and handled.

### Determination of relevant physiological and biochemical indices

Soluble sugars, glucose, and fructose, were analyzed as described by Abbaset al., 2024; El-Mahdy et al., 2024 [97, 109].The quantification involved two distinct methodologies. The first method, adapted from Sonnewald et al. (1991) [110] and Ortiz-Marchena et al. (2014) [111], involved extracting leaf samples from all tomato wild-type (control) and accessions under various concentrations of NaCl (treatments) using 80% ethanol in 10 mM HEPES-KOH (pH 7.7) at 80 °C for 2 h. The supernatant was utilized to measure soluble sugars, glucose, and fructose concentrations through the sequential addition of specific enzymes—5 units each of glucose-6-phosphate dehydrogenase and hexokinase, 2 units of glucose-6-phosphate isomerase, and 20 units of invertase—followed by the monitoring of NAD^+^ reduction at 340 nm absorbance at various intervals. Starch granules were isolated using a modified approach based on the method by Ortiz-Marchena et al. (2014) [111]. Three biological replicates were performed for each indicator. Furthermore, the levels of chlorophyll a, b, and total (a + b), were determined according to the modified method by El-Mahdy et al., 2024 [97]. Briefly, about 20–30 mg of fresh leaf tissues were collected from wild and treatment *S. lycopersicum* plants, and then each sample was added into a centrifuge tube with 4 mL dimethylformamide (DMF) and kept in the dark overnight to prevent chlorophyll degradation. The levels of chlorophyll a, b, and total (a + b) in the extracts were completed using the method described by El-Mahdy et al., 2024 [97]. The absorbance of the extracted chlorophyll was measured at wavelengths of 664 and 647 nm using JENWAY 6505 UV/Vis spectrophotometer.

### Statistical analysis

In this study, we utilized an analysis of variance (ANOVA) to compare the mean values of physiological and biochemical parameters obtained from two Egyptian tomato genotypes before and after treatment with various concentrations from NaCl. Moreover, the trial was carried out based on a completely randomized design (CRD) with three replications, and evaluate disparities in physiological and biochemical content (e.g., soluble sugars, glucose, fructose, total chlorophyll, chlorophyll a, and chlorophyll b) based on the type of genotypes and the treatment of plantlet with various concentrations from NaCl. Expressive statistics were further explored through analysis of variance (ANOVA) of SPSS 21.0 software (IBM Corp., 2020; Abbas et al., 2024) [109, 112]. Analysis of variance (ANOVA) was performed applying, followed by Duncan’s multiple range tests. Significance levels were indicated as (*) for P-values less than 0.05, (**) for *P* < 0.01, (***) for *P* < 0.001, and (****) for *P* < 0.0001, demonstrating the highest degree of significance. This allowed us to identify the tomato genotypes, time and NaCl concentrations that exhibited statistically significant differences in the content of soluble sugars, glucose, fructose, total chlorophyll, chlorophyll a, and chlorophyll b.

## Electronic supplementary material

Below is the link to the electronic supplementary material.

Supplementary Material 1

## Data Availability

Data is provided within the manuscript or supplementary information files.
